# A Cross-Sectional Study on the Presence of Mamelons in Northeastern India

**DOI:** 10.7759/cureus.74880

**Published:** 2024-11-30

**Authors:** Anamika Nath, Saindhya Sonowal, Pawan Kumar, Ispita Das

**Affiliations:** 1 Forensic Medicine, Tezpur Medical College & Hospital, Tezpur, IND; 2 Dentistry, Nagaon Medical College and Hospital, Nagaon, IND; 3 Periodontics, Dentocare Superspeciality Dental Clinic, Tezpur, IND; 4 Department of Forensic Odontology, Jagadguru Sri Shivarathreeshwara (JSS) Dental College and Hospital, Mysuru, IND

**Keywords:** age estimation, cross-sectional, dental anthropology, forensic odontology, incisors, mamelons

## Abstract

Introduction

Dental anthropology plays a pivotal role in human evolution and forensic sciences. This study explores a unique method for age estimation-analyzing mamelons on incisors. Mamelons are small projections on the incisal edge of permanent incisors, exhibiting age-related changes. This departure from conventional methods presents an innovative and potentially efficient approach to age estimation.

Materials and methods

A cross-sectional study was conducted in two tertiary care medical institutes and one private clinic in northeastern India over three months. Participants from the community, selected through convenient sampling, underwent data collection on demographic and habit-related variables. The sample size was 1000 teeth, and the age of participants was 5-40 years. Descriptive statistics were used to summarize the demographic and habit-related variables. The primary statistical analysis involved Pearson correlation tests to explore the relationships between categorical, demographic, and habit-related variables. The presence or absence of mamelons on incisors was determined using intraoral examination and study casts. Statistical analysis was performed using Python software.

Results

A total of 60.8% of teeth showed the presence of mamelons. Typical Three Mamelon Configurations with lobes of similar size constituted the majority (53%). Coefficients were statistically significant in age groups 5-7 years (0.5407), 8-10 years (0.5579), and 11-13 years (0.5476). Malocclusion could lead to the retention of mamelons even in higher age groups.

Conclusion

Estimating age through mamelons on incisors is a promising avenue in dental anthropology and forensics. Despite the need for further validation and research across diverse populations, its non-invasiveness and forensic applications make it a valuable addition to age estimation methodologies. The presence of mamelons showed a significant reduction with age and was significantly found in tribal communities. However, there was no strong relation with sex, diet, or brushing habits.

## Introduction

Dental anthropology has long been an invaluable field in the study of human evolution and forensic sciences. In recent years, researchers have delved into innovative techniques for age estimation, aiming to refine and improve existing methods. One such intriguing avenue of study is the estimation of age through mamelons on incisors - an unconventional yet promising approach. Mamelons refer to small projections located on the incisal edge of permanent incisor teeth [1]. When a new incisor emerges, it displays protuberances on the incisal edge, forming distinct mamelons separated by grooves. All anterior teeth exhibit four lobes-three on the labial side and one on the lingual side [2]. Each labial lobe concludes with a rounded elevation known as a mamelon. The prominence of mamelons is most evident in newly erupted permanent central maxillary incisors. Typically, these mamelons diminish with age due to wear and tear, resulting in a flat tooth surface [3]. This phenomenon is observed in both maxillary and mandibular incisors.

Mamelons on the maxillary central incisors vary in size [4,5]. The mesial mamelon has an elevated shoulder, the middle one is the smallest, and the distal mamelon has a lower shoulder. In contrast, the mandibular central incisors also possess mamelons, but the mesial and distal mamelons are equally prominent. Primary dentition lacks mamelons, serving as a distinguishing factor between permanent and deciduous dentition. Additionally, mamelons can persist and become more pronounced in specific syndromes and conditions, such as KBG syndrome and microcephalic osteo-dysplastic primordial dwarfism [6,7]. Notably, mamelons consist solely of enamel extensions without underlying dentin. This composition, coupled with their thinness, imparts translucency and enhances their visibility [8].

Using mamelons as a marker for age estimation represents a departure from conventional methods, providing an alternative approach that may prove to be both accurate and efficient. Although there have been very few studies on the presence of mamelons in different ethnicities and age groups, there is none in the northeastern zone of the nation. The objective of the study was to find the presence of mamelons in permanent incisors in the northeastern population of India in different age groups and to see the effect of sex, ethnicity, diet, and brushing habits on the presence of mamelons.

## Materials and methods

Study design

This is a cross-sectional study done at two tertiary care medical institutes and one private clinic in northeastern India conducted for 3 months.

Study participants

The study included patients coming to these centers for complaints not related to incisors and were willing to participate in the same in the age group of 5-40 years. The sampling method was convenience sampling. Those with deformity or disease on the incisor teeth were excluded from the study. A total of 1000 teeth as a sample were included in this research.

Data collection and ethical consideration

Upon obtaining ethical approval from the Institute Ethics Committee vide IEC Sl. No: 066/2023/TMCH, dated 09/09/2023, and informed consent from the study participants or their legal guardians (if the participants were minors), trained co-investigators conducted interviews on demographic variables and habit-related variables with the participants.

Variables

Demographic Variables

Age (categorized as 5-7 years, 8-10 years, 11-13 years, 14-16 years, 17-19 years, 20-22 years, 23-25 years, 26-28 years, 29-31 years, 32-34 years, 35-37 years, 38-40 years), Sex (Male, Female, Others), Ethnicity (Assamese, Tribal, Bengali, Tea-Tribe).

Habit-Related Variables

Dietary habits (Vegetarian, Non-Vegetarian), Brushing habits (Does not brush, Brushes once, Brushes twice, Brushes more than twice)

Outcome Variable

Presence or absence of mamelons on the incisor teeth

Armamentarium

Mouth mirror, UNC -15 periodontal probe (Hu-Friedy, Chicago, USA), tweezer

Materials Used for Fabrication of Study Cast

Alginate impression material (DENTSPLY India Pvt. Ltd., Haryana); dental stone (Kalstone, Kalabhari Karson Pvt. Ltd, Mumbai).

Study procedure

**Figure 1:**
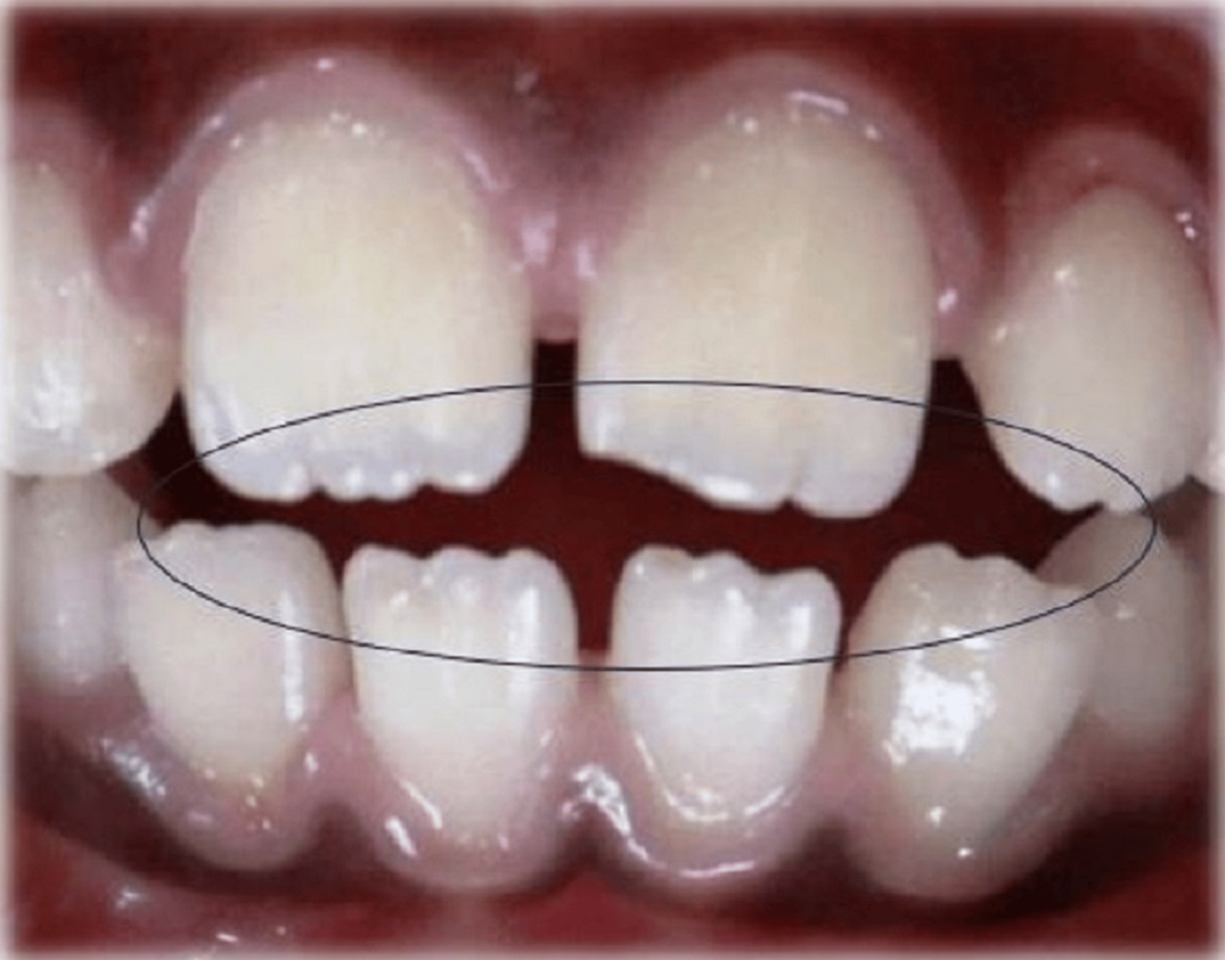
Mamelon expression on teeth.

The pattern of mamelons in maxillary and mandibular central and lateral incisors was noted intraorally using Fitzgerald 12 graded scoring criteria (Figure 1) [9].

**Figure 2:**
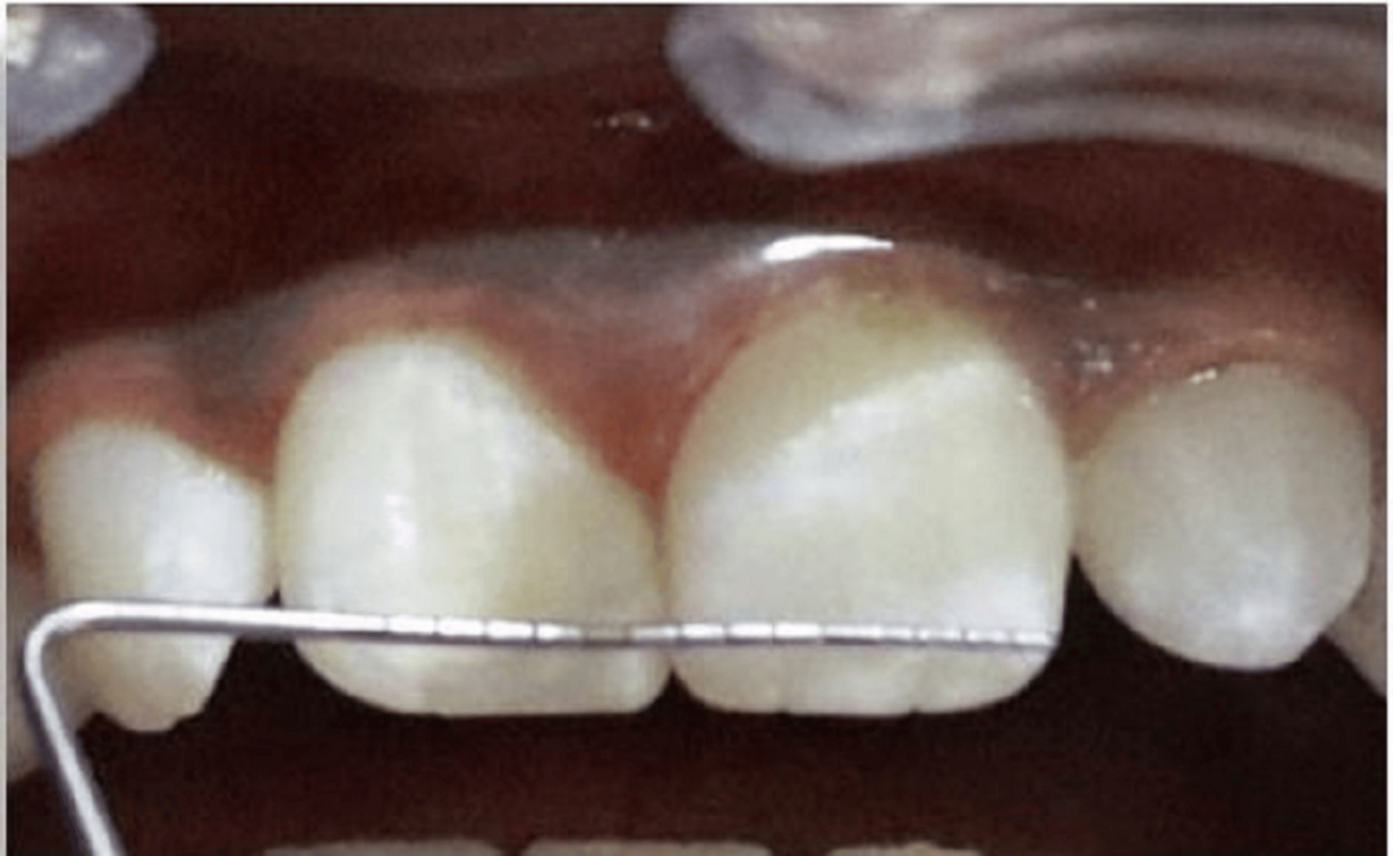
Measuring the size of Mamelons using UNC Probe 15.

The data recorded were categorized into different age groups of the subjects. Dental Impressions of the maxillary and mandibular teeth of all the subjects were recorded. These alginate impressions were poured with dental stone. These casts obtained were then screened for the presence of mamelons and their corresponding size was noted using a UNC -15 periodontal probe (Figure 2).

Data analysis

The collected data was analyzed using statistical software Python (v3.9), SciPy (v1.7.1), and statsmodels (v0.14.0) [8,9]. Descriptive statistics were used to summarize the demographic and habit-related variables. The primary statistical analysis involved Pearson correlation tests to explore the relationships between categorical, demographic, and habit-related variables.

## Results

Out of 125 study participants, 61% of the participants had mamelons. Eight numbers of incisors per participant were considered for the study constituting a total of 10000 teeth. The incisors exhibited Typical Three Mamelon Configuration (53%), Distal Notch shape (19%), and Median Dominance (13%) being common as depicted in Table 1.

**Table 1:** Count of Shape of Mamelons.

Shape of Mamelons	Count
Distal notch	68
Median dominance	49
Median dominance with caniniform incisal edge	1
Median notch	25
Mesial notch	6
Straight incisal edge with no evidence of mamelon	3
Three mamelon configuration with weak expression & tapering distal crown contour	19
Typical three mamelon configuration with lobes of similar size	194

Mamelon presence showed teeth 11, 21, 22, 32, 41, and 42 having 13-16%, while teeth 12 and 31 were at 2.9% and 5.1%, respectively as shown in Table 2. 

**Table 2:** Tooth Number Distribution with the Count of Mamelons.

Tooth Number	Count of Mamelons
11	47
12	9
21	43
22	46
31	17
32	39
41	51
42	37

Notably, 66% of mamelons were in patients under 19 years old. Regression analysis showed an increase in mamelon probability for age groups 5-7, 8-10, and 11-13 years, the coefficient decreased for age groups 14-16 and 17-19 years significant negative effect in the probability of the presence of mamelons from age groups starting 20 and onwards. No significant effect was found for age groups 20-25 years, while those 26 years and older exhibited a significant negative effect on mamelon presence, suggesting a decline in prevalence in adults. Sex does not significantly impact mamelon presence, as both males and females exhibit similar probabilities. Ethnicity plays a role, with tribal communities showing a higher likelihood of having mamelons (p = 0.04) compared to other ethnic groups, suggesting a genetic factor. However, diet (vegetarian vs. non-vegetarian) and brushing habits (once or twice daily) do not significantly affect the likelihood of mamelons. Overall, age and ethnicity, particularly tribal origin, are the most influential factors, while sex, diet, and brushing habits show no meaningful correlation with mamelon presence (Table 3).

**Table 3:** Regression Analysis of Age Groups, Sex, Ethnicity, Dietary Habits, and Brushing Habits with Respect to the Presence of Mamelons.

Variables	Coefficient	Std. Err.	p-value	CI 0.025	CI 0.975
Age Group in Years					
5-7	0.5407	0.089	0	0.364	0.717
8-10	0.5579	0.101	0	0.359	0.757
11-13	0.5476	0.114	0	0.323	0.772
14-16	0.3091	0.142	0.03	0.03	0.589
17-19	0.3113	0.107	0.004	0.101	0.522
20-22	-0.1567	0.059	0.008	-0.273	-0.041
23-25	-0.1869	0.063	0.003	-0.311	-0.063
26-28	-0.4289	0.078	0	-0.582	-0.276
29-31	-0.3689	0.066	0	-0.498	-0.24
32-34	-0.2215	0.092	0.017	-0.402	-0.041
35-37	-0.4827	0.084	0	-0.648	-0.318
38-40	-0.4696	0.085	0	-0.638	-0.301
Sex					
Female	-0.0058	0.097	0.952	-0.198	0.186
Male	-0.0427	0.096	0.655	-0.231	0.146
Ethnicity					
Assamese	0.2065	0.146	0.158	-0.081	0.494
Bengali	0.2969	0.183	0.106	-0.064	0.658
Tribal	0.3043	0.147	0.04	0.013	0.595
Dietary Habit					
Non-Vegetarian	-0.0489	0.116	0.673	-0.277	0.179
Vegetarian	0.0003	0.12	0.998	-0.236	0.236
Brushing Habits					
Does not Brush	0.2816	0.373	0.451	-0.453	1.016
Brushes Once Daily	0.3356	0.364	0.357	-0.382	1.053
Brushes Twice Daily	0.4136	0.369	0.263	-0.314	1.141

**Figure 3:**
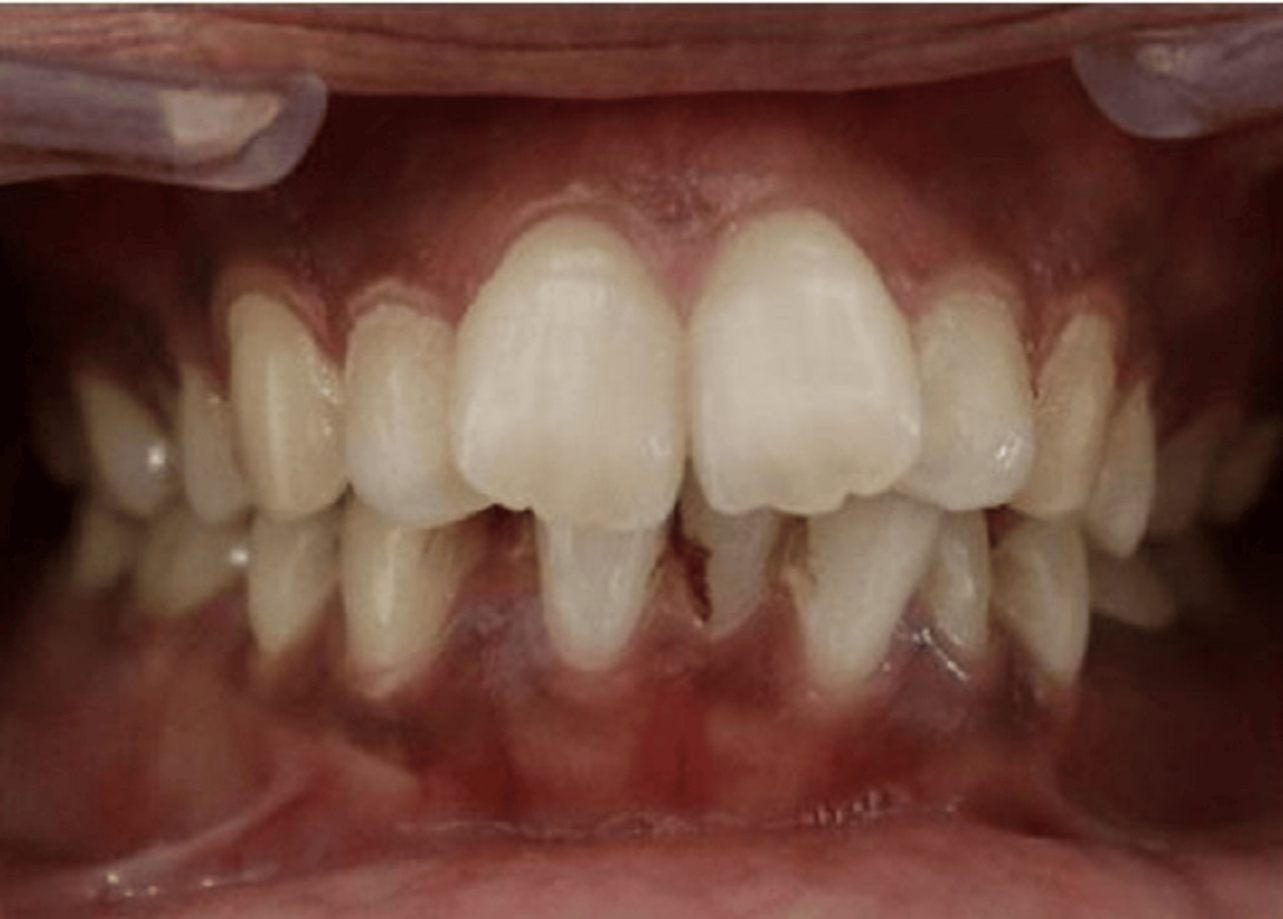
Malocclusion causing retainment of mamelons in higher age groups.

Some participants in higher age groups exhibited mamelons who had come for treatment for malocclusion and were majorly females (Figure 3).

## Discussion

The estimation of age through mamelons offers several advantages over traditional methods. Firstly, it is a non-invasive procedure that can be conducted without the need for complex equipment, making it highly accessible in both clinical and forensic contexts. This method is particularly useful in cases where traditional age estimation techniques, such as dental attrition or bone ossification, may be less reliable due to factors like malnutrition or systemic disease. Furthermore, mamelons provide a reliable estimate of age, especially in younger individuals where these structures are more pronounced and intact. One of the major benefits of this technique is its applicability in situations where more advanced diagnostic tools may not be available, such as in field-based forensic work or under-resourced regions. However, challenges exist in the application of this technique. Individual variations in dental development, occlusal relationships, and lifestyle factors such as diet, oral hygiene, or parafunctional habits like bruxism can influence the rate at which mamelons wear down [10]. Research conducted by Chegini-Farahani et al. found that 90% of all incisors had mamelons, but differences were observed in the shape and prominence between mandibular and maxillary arches, with notable variations attributed to ethnicity [10]. Our research revealed that 61% of teeth exhibited mamelon expression, indicating a relatively high prevalence. Typically, mamelons are not observable in the permanent dentition of adults, as they tend to wear off once the tooth engages in functional contact with its opposing counterpart, a process thoroughly documented in dental literature [3]. The percentage of cases displaying mamelons gradually decreases with each successive decade of life, suggesting a clear correlation with natural wear over time [11]. Mamelons are more prominently visible on the central incisors compared to the lateral incisors, likely due to differences in functional engagement [12]. Interestingly, in situations where there is an anterior open-bite relationship between the maxillary and mandibular teeth, mamelons may persist into adulthood, as there is no functional contact between the teeth to cause wear [11]. This phenomenon has also been observed in cases of malocclusion, such as in patients with anterior open bites, where mamelons are preserved into later life (Figure 3) [13]. Additionally, patients with parafunctional habits like tongue thrusting or mouth breathing may show prolonged retention of mamelons due to reduced occlusal contact [14].

Our study is consistent with previous research showing the persistence of mamelons in females of higher age groups [12]. This observation may be related to differences in bite force and occlusal patterns, as well as other physiological factors influencing dental wear. Other studies have supported the hypothesis that gender, occlusion, and functional usage of teeth significantly impact the rate of mamelon wear, providing further insight into the importance of mamelons as indicators of dental and occlusal health [15].

The limitation of the study is that the convenience sampling method was used. Participants visiting the two tertiary care medical institutes and one private clinic were included. The strength of the study is that it is new in the northeastern zone of the nation. It is reproducible in similar participants and settings.

## Conclusions

The estimation of age through mamelons on incisors presents a novel and promising avenue in the field of dental anthropology and forensic sciences. While further research and validation are necessary to establish the accuracy and reliability of this technique across diverse populations, its non-invasive nature and potential applications make it a valuable addition to the toolkit of forensic experts and dental professionals. As advancements in technology and research continue, the estimation of age through mamelons may become a mainstream method, contributing to our understanding of human history and aiding in forensic investigations. The presence of mamelons showed a significant reduction with age and was significantly found in tribal communities. However, there was no strong relation with sex, diet, or brushing habits.
